# In a Bulgarian Hospital

**Published:** 1913-01-04

**Authors:** 


### IN A BULGARIAN HOSPITAL.

### AN EXPERIENCE AT THE FRONT.

[We have received the following letter from a correspondent at the front. The censorship is so strict that ingenuity was taxed severely to get this through at all.]

In a beautiful village four miles from one of the larger towns the Bulgarian Government has set apart some large buildings for a hospital for sick and wounded Turks. A detachment from the Red Crescent Society in London has been doing fine work amongst them, and although there were but two doctors and four dressers at the beginning, there were sometimes as many as twelve operations to be performed in the day, and, indeed, night, as work had to be carried on till after midnight, and by the light of a miserable paraffin lamp. Many cases were in a pitiable state, the inevitable result of the hardships and suffering the defeated Turks underwent. However, the humane behaviour of the Bulgarian authorities in sending their sick prisoners to a singularly healthy place blessed with a magnificent and pure water supply has, added to the naturally hardy character of the Turks, kept the death-rate very low. Very remarkable recoveries have been witnessed by the two English surgeons with great satisfaction. Coming out to Bulgaria to see if I could be of any use, I have had the privilege of being attached—the only trained nurse—to the hospital, and the work is peculiarly interesting.

The language difficulty is hampering, for while the upper officials speak French as a rule, the lower classes (from which the orderlies are taken) rarely speak anything but Bulgarian. Then, of course, the patients speak various Turkish dialects, and there are besides the Albanians, Greeks, Arabs, and other races, who cannot understand one another. But they are docile and grateful, and are in no hurry to be discharged, as that means inferior diet and prison. Every class is represented, from a negroid type to a courtly person from Stamboul. Some through all the hardships of the last weeks have kept themselves clean and tidy; others, unfortunately the majority, arrive in a wretched state of dirt. Soap and water are appreciated by most of them, but owing to the state of their clothing thorough cleanliness is impossible.

How different the wards look from those of an English hospital! They are large and fairly well lighted; each bedstead has a straw mattress and pillow, sheet and pillow-cover, and a woollen rug: not content with this, most of the patients wear over their white cotton pyjamas as many coats and waistcoats as they can manage, all the colours of the rainbow, and every head is covered with a fez or turban, or sometimes just a coloured handkerchief. When convalescent they may be seen sitting cross-legged on their beds, the more devout praying at stated intervals, turned towards Mecca. As some weeks have passed since the heavy fighting took place, the majority of daily admissions are now medical cases—pneumonia, dysentery, malaria, and such diseases as are the consequences of exposure and starvation. It is hard not to be able to speak sympathetically to these poor prisoners in a strange land, and I wonder when peace comes how many will go home and find wife and children waiting for them.

